# Immunosenescence in Sepsis: Molecular Mechanisms and Potential Therapeutic Targets

**DOI:** 10.14336/AD.2025.0039

**Published:** 2025-03-11

**Authors:** Heyang Sun, Qianya Hong, Chenning Li, Shuainan Zhu, Ying Yu, Hao Zhang, Kefang Guo

**Affiliations:** ^1^Department of Anesthesiology, Zhongshan Hospital, Fudan University, Shanghai 200032, China.; ^2^Shanghai Key Laboratory of Perioperative Stress and Protection, Shanghai 200032, China.

**Keywords:** Sepsis, immunosenescence, cellular senescence, inflammation, aging, therapeutic targets

## Abstract

Sepsis is a systemic inflammatory response triggered by infection that can result in immune regulation disruption and organ dysfunction. As an individual age, a phenomenon known as immunosenescence occurs. Immunosenescence is characterized by the deterioration of immune cells and organs. This decline leads to immune dysfunction, which is closely associated with an increased mortality rate among elderly sepsis patients. Recent studies have revealed that various responses during sepsis can induce premature aging of immune organs and cells in younger individuals, a process referred to as premature immunosenescence, which further accelerates the progression of sepsis and contributes to adverse outcomes. There is a significant correlation between immunosenescence and sepsis; therefore, understanding the specific manifestations and underlying mechanisms of immunosenescence induced by sepsis is imperative. Additionally, treatment strategies aimed at reversing or alleviating both immune aging and immune suppression in septic patients are worthwhile exploring and will also improve understanding of the concept of immunosenescence.

## 1. Introduction

Sepsis represents a systemic inflammatory response elicited by infection. It is frequently observed in patients with severe trauma or infectious diseases [1, 2] and eventually causes disruption of inflammation and immune regulation in the body.

Research has indicated that the immune system deteriorates with advancing age in older adults who experience typical age-related changes. Physiologically, cellular senescence accompanies aging and is a major feature of aging [1]. Immune cells, as key regulators of cellular senescence [2, 3], undergo changes that lead to dysfunction during aging. This malfunction of the immune system leads to unsatisfactory vaccination performance; increased vulnerability to infections, age-related diseases, inflammatory diseases and malignancies; and delayed wound healing. This phenomenon involving disruption and remodeling of immune organ architecture and impaired functionality of both the innate and adaptive arms of the immune system is termed immunosenescence. Immunosenescence is characterized by several key features, including thymic involution, disruption of the balance between naive and memory T cells, an altered CD4:CD8 T-cell ratio, defective calcium signaling [4], alterations in epigenetics, and metabolic dysregulation [5]. The two central aspects of immune aging are the unbalanced inflammatory state and the degeneration of the immune system [6].

Research has revealed that several organs and cells age during sepsis and exhibit some senescent phenotypes [7-10], confirming that sepsis can induce premature aging in the human body. Moreover, the typical features of excessive inflammation and decreased immune function also occur in sepsis [11-13]. Multiple mechanisms induce inflammation, and mediators released from tissue and organ damage caused by inflammation are also involved in the inflammatory response and recruit many immune cells, resulting in a positive feedback loop to induce excessive inflammation. This excessive inflammation not only directly destroys tissues and organs but also impairs the immune function of the body, even directly causing immune cells to prematurely enter an aging state, which is marked by an increase in the discharge of anti-inflammatory mediators, increased programmed cell death of immune cells, T-cell exhaustion, cellular reprogramming via epigenetic alterations, decreased expression of activated cell surface molecules, and the generation of multiple suppressive cells, ultimately leading to immune system failure and decreased resistance to infections in patients with sepsis. The human body continually grows and develops, and these processes are influenced by life, diet, the environment, disease and other factors that can lead to changes in morphological structures and functions in the body and organs earlier than with natural aging-related degeneration; these changes are typical manifestations of premature aging [14-16]. Sepsis-induced immune degeneration that occurs prematurely in nonelderly patients can be thought of as disease-induced premature senescence of the immune system, i.e., premature immunosenescence.

Studies of immunosenescence in sepsis have become increasingly sophisticated in recent years. This review describes the manifestations of immunosenescence in nonelderly and elderly sepsis patients, explains the specific mechanisms by which representative cells of the innate and adaptive immune systems cause immuno-senescence, and presents relevant measures currently approved for clinical use that may be applied to treat or alleviate immunosenescence and immunosuppression according to the above mechanisms. Overall, this review further expands the current understanding of immunosenescence.

## 2. Immunosenescence in Sepsis

### 2.1 Sepsis-Induced Premature Immunosenescence

**Figure 1.**
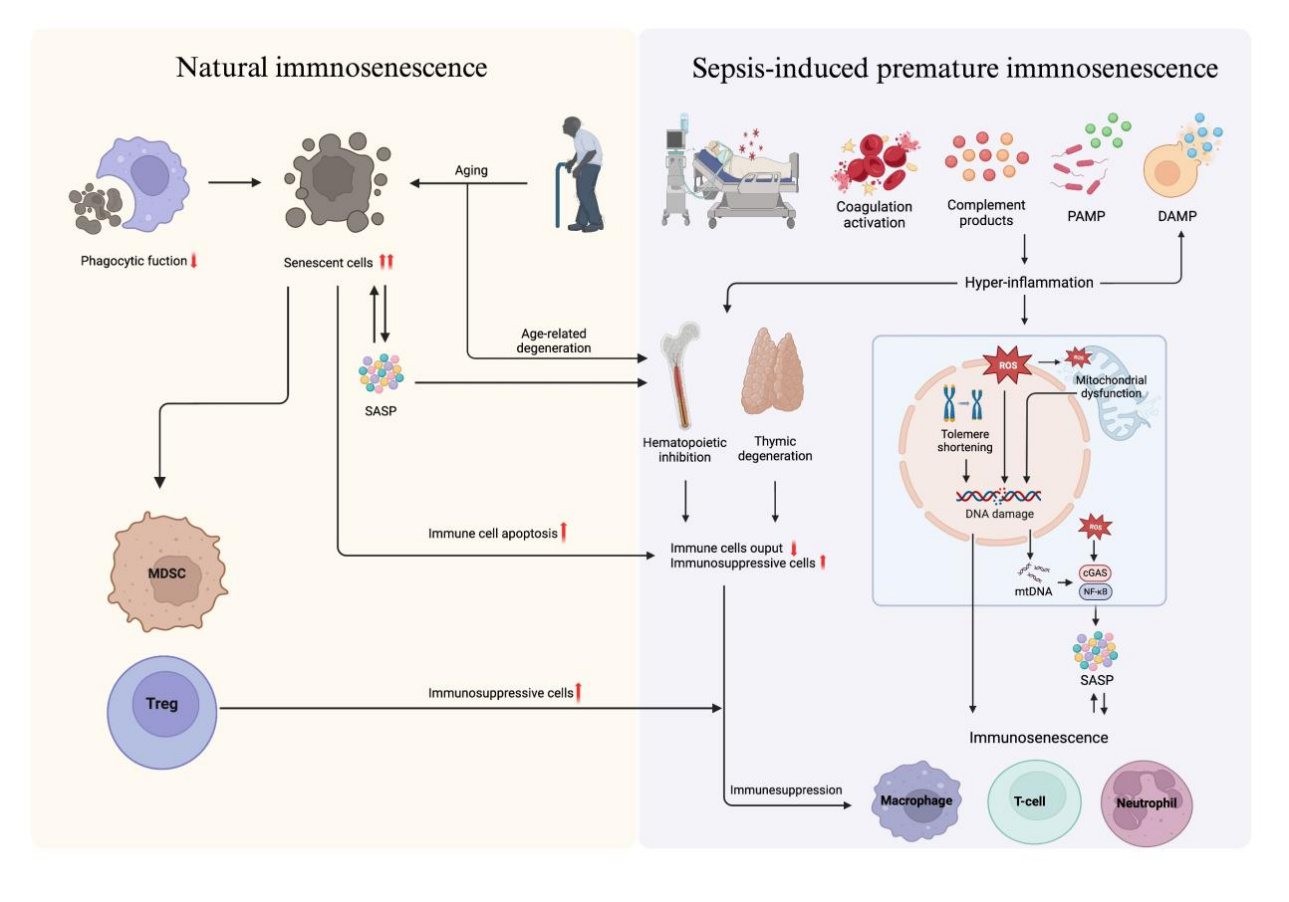
**Comparison of natural and sepsis-induced premature immunosenescence**. In sepsis, natural immune cells recognize PAMPs, activating the coagulation and complement systems and triggering an inflammatory response that causes tissue damage. Injured tissues release DAMPs, which are recognized as foreign by the immune system, further exacerbating inflammation. The uncontrolled inflammatory environment recruits more inflammatory cells and promotes inflammatory mediator production, creating a positive feedback loop. Excessive inflammation leads to telomere shortening, increased ROS, DNA damage, and mtDNA release, activating the cGAS/NF-κB pathway and producing SASP, leading to immunosenescence. ROS also directly participate in SASP production and mitochondrial dysfunction, causing further DNA damage. Inflammatory stimuli lead to HSC functional depletion, hematopoietic suppression, decreased thymic output, and increased number of immunosuppressive cells, collectively contributing to immune senescence. In elderly individuals, increased numbers of senescent cells and decreased phagocytosis impair senescent cell clearance and accumulation, increasing SASP secretion and creating a chronic inflammatory environment. Elderly individuals also have more suppressor cells and reduced bone marrow and thymus function. These changes further accelerate immunosenescence in sepsis patients and lead to worse prognosis in older adults with sepsis (PAMPs, pathogen-associated molecular patterns; DAMPs, damage-associated molecular patterns; ROS, reactive oxygen species; mtDNA, mitochondrial DNA; NF-κB, nuclear factor-κB; SASP, senescence-associated secretory phenotype; HSCs, hematopoietic stem cells).

Recent studies have shown that sepsis induces cellular senescence in multiple organs, including the liver [9], kidney [8], and heart [10], and the upregulation of senescence-related genes has been detected in relevant cells [9], indicating that sepsis is crucial for inducing premature senescence in the body and that immunosenescence is a core component of aging. Sepsis disrupts the body's immune balance by triggering a complex, pro- and anti-inflammatory response that impairs homeostasis [11]. The body's initial line of defense involves natural immune cells that recognize pathogens or pathogen-associated molecular patterns (PAMPs) to initiate an immune response. The complement system is subsequently activated, which releases C3a and C5a with potent proinflammatory activity and promotes the mobilization and activation of leukocytes, endothelial cells, and platelets. The tissue is damaged as a result of this process, which prompts the release of damage-associated molecular patterns (DAMPs), which are subsequently recognized by the immune system as foreign substances and further promote inflammation (Fig. 1) Under conditions of uncontrolled inflammation, inflammatory cells are persistently recruited, which further promotes the generation of inflammatory mediators, establishing a positive feedback loop that exacerbates inflammation and culminates in an overactive immune reaction known as a “cytokine storm.” This process results in widespread tissue damage and multiorgan failure and potentially contributes to the deterioration of the immune system over time. Studies have revealed that a variety of sterile and infection-associated inflammatory stimuli stimulate the transition of primitive hematopoietic stem cells (HSCs) from a prolonged quiescent state to active proliferation [7]. The downregulation of CD201 on the surface of HSCs promotes myeloid escape during emergency granulopoiesis in the early phase of LPS-induced acute infection [17], which is consistent with the changes observed in HSCs during the progression of senescence [18]. Inflammation-associated stress-induced hematopoiesis leads to a reduced repopulation potential of HSCs after transplantation, resulting in long-term inhibition of hematopoiesis due to the progressive and irreversible depletion of functional HSC pools [7], resulting in various cellular and molecular characteristics associated with accelerated aging. Additionally, thymic degeneration occurs in patients with sepsis, leading to reduced thymic output, which is engaged in the development of lymphopenia and anti-inflammatory response syndromes and accelerates the process of immunosenescence [19]. Severe or persistent lymphopenia in patients with sepsis is associated with increased mortality [20]. Lymphocytopenia has been shown to be independently associated with a higher 28-day mortality rate in patients with sepsis [21]. In a cohort study of 401 children with severe sepsis, 38% of the children presented persistent lymphopenia, which was associated with a composite outcome of prolonged MODS or PICU mortality. Notably, this persistent lymphopenia remained unresponsive to reversal during the study period [22]. IL-7 has been demonstrated to reverse sepsis-induced lymphopenia and to augment T-cell proliferation and activation [23]. The lymphopenia that occurs in sepsis is not caused unilaterally by thymic degeneration; sepsis-induced lymphocyte apoptosis is also involved in this process [24]. The importance of the pathophysiology of augmented apoptosis in sepsis has been demonstrated in animal models, and interventions that inhibit apoptosis improved survival [12]. Recent studies have identified a U-shaped correlation between the lymphocyte count and the risk of death in hospitals among patients with sepsis. The lowest risk was observed at moderately elevated levels, indicating that both lymphopenia and extreme lymphocytosis are associated with a poorer prognosis [25]. This discrepancy may be related to the heterogeneity of the study population (e.g., age, underlying immune status) or the lack of lymphocyte subset analysis. These findings further underscore the importance of the delicate balance between immune impairment and excessive inflammation in patient prognosis. It is imperative to acknowledge the limitations inherent in therapeutic interventions and to prioritize their judicious application.

Changes in other systems can also lead to increased susceptibility to sepsis. The gut is an important immune organ in which trillions of symbiotic bacteria are colonized, which play a role in maintaining homeostasis and defending against pathogen invasion of the host. The microbiota directly competes for nutrients, maintains epithelial barrier function, produces antimicrobial peptides, and regulates the production of antimicrobial proteins by host cells [13], and the disruption of intestinal homeostasis increases the probability of sepsis in patients [26]. Notably, lncRNA H19 is significantly upregulated in the human intestinal mucosa of sepsis patients, compromising the effectiveness and integrity of the mucosal barrier function of the small intestine [27], and this barrier function is crucial to consider in the discourse on immunosenescence, particularly in the context of elderly individuals. Furthermore, critically ill patients in the surgical intensive care unit (ICU) frequently present an imbalance in glucose homeostasis, which is a consequence of the injury stress response and persistent proinflammatory response. The failure of glycemic control is closely associated with adverse prognoses, including death, nosocomial infection, prolonged ICU stay, and critical illness neuropathy [28]. During the pathological process of sepsis, intestinal L cells secrete an enteric incretin hormone, GLP-1, and there is an early surge in compensatory GLP-1 expression [29]. As the disease progresses, this regulatory system is suppressed, and the level of endogenous glucagon increases abnormally. These changes exacerbate stress hyperglycemia and the systemic inflammatory response and reduce the ability to resist pathogen invasion [30]. Accordingly, artificially administered exogenous GLP-1 receptor agonists (GLP-1 RAs) have been demonstrated to function through a dual mechanism. On the one hand, they have been shown to reduce blood glucose variability by optimizing blood glucose control. On the other hand, they have been demonstrated to improve the prognosis of sepsis through their multiorgan protective effects. This provides a new direction for clinical intervention [31]. However, it is important to note that clinical trials have reported side effects such as gastrointestinal problems in some patients treated with GLP-1 RA, suggesting that its efficacy may be affected by the timing of administration and basal metabolic status [32]. Notably, elevated GLP-1 levels within 24 hours of sepsis have been demonstrated to be strongly associated with early death and serve as a predictor of early death or persistent organ dysfunction. Among early survivors, persistently elevated GLP-1 levels on day 14 have been shown to strongly predict death or severe dysfunction at 6 months. Persistently elevated GLP-1 levels may serve as a marker of a nonresolving catabolic state and are associated with muscle wasting and adverse outcomes following sepsis and chronic critical illness.

Prolonged exposure to microbial challenges or pathogenic microorganisms accelerates the onset of cellular senescence and induces premature aging of immune cells [33]. Elevated levels of senescence-associated secretory phenotype (SASP) markers, including IL-6, IL-1β, and TNF-α, have been detected in mice subjected to cecal ligation and puncture (CLP) to induce sepsis [9]. Dysregulation of immune cell metabolism in a proinflammatory microenvironment results in impaired NAD+ metabolism and increased immune cell senescence and SASP [34]. The inflammatory context of SASP and sepsis is linked to mitochondrial dysfunction and increased production of reactive oxygen species (ROS), which fosters the onset of cellular senescence [35]. Sepsis induces the shortening of telomeres [36], which are critical for safeguarding genomic integrity against nuclear degradation, the DNA damage response, and unwanted DNA recombination damage in eukaryotes. Shortening of telomeres leads to an inability to bind sufficient telomere capping proteins, which function as exposed DNA ends and activate the DDR pathway. Concurrently, short telomeres maintain an adequate quantity of telomere-binding proteins, which possess the capacity to impede DNA repair and evade fusion, thereby generating persistent DNA damage signals and fostering cell senescence [37]. Although telomere shortening is one of the earliest and most characteristic mechanisms induced by cell senescence, sustained DDR activation of telomeres can occur both during telomere shortening in proliferating cells and during telomere DNA damage in nonproliferating cells (resting or terminally differentiated), indicating that it can occur independently of changes in telomere length [38]. Given the established relationship between telomere shortening and numerous diseases, the potential influence of other diseases on the state of sepsis and telomere length cannot be excluded [37]. Nevertheless, the correlation between telomere shortening and sepsis has been definitively demonstrated. It has been observed that telomere length tends to decrease further as sepsis progresses [39]. In related studies, septic patients with shorter peripheral blood leukocyte telomeres presented a lower 90-day survival rate, which was closely related to a decrease in the survival rate and more severe ARDS in critically ill patients [40]. Furthermore, the hypothesis that telomere dysfunction is the primary cause of aging and age-related diseases may not be entirely accurate. Instead, the activation of DDR resulting from telomere dysfunction may be the primary factor in the development of cellular senescence. The subsequent propagation of aging signals through the SASP is a consequence of DDR and cellular senescence [41]. DDR can also be induced by conditions other than telomere shortening. The aggravation of mitochondrial damage in sepsis leads to the excessive production and accumulation of ROS. The increased production of ROS has been demonstrated to trigger the NF-kB transcription factor cascade, which subsequently contributes to the synthesis of proinflammatory cytokines [42]. ROS levels that exceed the threshold required for cellular signaling and overwhelm the detoxification capacity of the biological system can lead to oxidative damage to various biomolecules, including DNA [43]. As telomeric DNA is highly sensitive to oxidative DNA damage [44], excessive ROS levels can thus cause DNA damage [45, 46], and the accumulation of such damage plays a pivotal role in promoting cellular senescence and driving the SASP [47], inducing senescence in both immune cells and those within the nonhematopoietic spectrum [48]. In addition, ROS and DNA damage can promote paracrine mechanisms to induce senescence in neighboring cells [49, 50]. However, differences in the presence of telomere dynamics in different immune cell subsets (e.g., T cells vs. monocytes) as well as the relative contribution of sepsis-associated oxidative stress (ROS) to telomere-dependent and nontelomere-dependent senescence (e.g., epigenetic alterations) remain to be elucidated. Inflammasomes, a class of cytoplasmic multiprotein oligomers, are involved in further induction of immunity and stimulation of inflammatory caspases [13], specifically caspase-1, which cleaves GSDMD, promotes the secretion of IL-1b and IL-18 and induces pyroptotic death in monocytes and macrophages [11]. C5a of the complement system inhibits neutrophil antimicrobial function by disrupting phagosome maturation through phosphoproteomic remodeling, causing immuno-deficiency in critical illnesses [51]. These findings highlight the mechanisms by which inflammation during sepsis can suppress immune function.

With the aggressive progression of the disease, immunodynamics enter a state of immunosuppression. Recent studies have revealed that sepsis patients experience immunosuppression at an early stage [52]. Long-standing evidence indicates that immunosenescence is driven primarily by increased functioning of immunosuppressive cells rather than solely by cellular senescence [53]. The phenomenon of cellular senescence is closely connected with the initiation of immunosuppressive networks, including immature myeloid-derived suppressor cells (MDSCs), and an enhanced function of regulatory T cells (Tregs) [53]. In sepsis survivors, immature myeloid-derived cells not only increase in number but also gradually develop MDSC-specific immunosuppressive functions associated with specific epigenomes over time [54]. The early proinflammatory response is closely associated with both tissue injury and impaired organ function. In contrast, simultaneous or delayed onset of an anti-inflammatory response significantly contributes to persistent immunosuppression, which increases the likelihood of nosocomial infections, rehospitalization rates, and potentially even mortality rates [12, 13].

In addition to the above factors, the immune system undergoes a decrease in both the functionality and quantity of immune cells themselves. The upregulation of key senescence-associated genes has been detected in T cells, neutrophils and macrophages from sepsis patients, confirming the occurrence of premature immune failure in sepsis patients [9, 55]. Sepsis results in a reduction in immune cell counts and functional capabilities, thereby impacting the innate and adaptive immune systems [11, 56]. Within 2 to 4 days of the onset of sepsis, lymphocyte apoptosis and substantial decreases in CD4+ and CD8+ T cells occur. Severe sepsis leads to the loss of precursor specificity in immature CD8+ T cells, thereby impeding their capacity to mount an immune response to novel antigens. This results in an immune deficiency in sepsis-related memory CD8+ T cells, characterized by a decrease in sensitivity to antigen recognition and cytokine secretion, as well as a reduction in cell proliferation and pathogen clearance [57]. A simultaneous tendency toward T-cell failure [58] leads to lymphopenia, which is linked to increased mortality risk and hospital-acquired infections among patients suffering from sepsis [59]. A number of studies have also demonstrated that the proliferative capacity of CD4+ and CD8+ T cells is not sustained for an extended period following suppression. Rather, it undergoes a gradual recovery within a one-month timeframe through various mechanisms, including homeostasis proliferation, antigen-driven proliferation in response to infection during sepsis, and other pathways. However, the body's capacity to eliminate pathogens is significantly diminished, and immune-related cytokines are substantially damaged [60]. Autopsies of both adults who died of sepsis [61] and children with sepsis-induced multiorgan failure [62] clearly revealed extensive loss of adaptive immune cells. This reduction is also related to the degeneration of the bone marrow and thymus mentioned above. Thymic atrophy is observed in septic mice [63] and is accompanied by a low homing capacity of T cells. In contrast, the differentiation of early T-cell lineage precursors in the bone marrow is consistently inhibited postsepsis and may also be related to the loss of thymic apoptosis. These findings indicate that systemic immunosuppression after sepsis affects primary and secondary lymphoid organs in addition to circulating cells. In contrast, specific RNA sequencing of lymphocytes and monocytes in the peripheral blood of septic and nonseptic patients has revealed that the number of differentially expressed genes in the monocytes of septic patients was approximately five times greater than that in the monocytes of CD8 cells, 55% of which were downregulated. Furthermore, the expression of monocyte costimulatory molecule genes (CD86 and OX40L) was reduced, and 10 immune response pathways (including the NF-AT, ICOS, and JAK-STAT pathways) were inhibited. The IL-10-mediated immunosuppressive pathway was activated, which severely impaired the normal function of the body's immune system [64]. Furthermore, monocytes in sepsis upregulate the expression of a significant number of cytokines, chemokines, cell surface molecules, and transcription factors. This upregulation results in the activation of the transcription factor NF-κB, which is central to inflammatory responses and plays an essential role in immune responses and inflammation [65]. However, HLA-DR expression has been reported to be 70% lower in septic patients than in nonseptic patients [11], whereas HLA-DR is pivotal in initiating adaptive immunity, indicating that monocytes are dysfunctional and act as biomarkers for immunosuppression [66]. Analysis of differential gene expressions in monocytes can help distinguish some cases of sepsis from nonseptic conditions [67]. This analysis can also serve as a metric for the evaluation of sepsis. Nevertheless, the diagnostic and therapeutic implications of this approach require further validation.

These findings offer additional support for the existence of early immunosenescence in the context of sepsis and its importance for the progression of sepsis.

### 2.2 Septic Manifestations of Natural Aging of the Immune System

Retrospective analysis of sepsis cases involving hospitalization from the NDCMS and NMSS databases between 2017 and 2019 revealed that of the 9,455,279 patients identified, 8.7% were babies younger than one year, 11.7% were children aged one to nine years, and 57.5% were elderly individuals aged 65 years and above [68]. This increase in infection rates with age is closely linked to age-related alterations found in older adults, and deterioration of the immune system is a key factor in this phenomenon. Senescent cells release a diverse array of proinflammatory factors [69], which are initially intended to recruit immune cells to eliminate senescent cells and promote renewal and tissue protection [47]. As the body ages, the functionality of the immune system decreases, resulting in reduced phagocytic clearance and the accumulation of many senescent cells [4, 5]. Atrophy of the thymus gland results in diminished functionality of B cells and T cells, consequently disrupting the equilibrium between proinflammatory and anti-inflammatory activities [1]. These factors promote the development of SASP, a condition characterized by chronic inflammation and a significant inflammatory hallmark of cellular senescence (Fig. 1). This systemic state of persistent inflammation, known as inflammaging, is considered a central part of immunosenescence [6, 70]. The inflammatory factors present in the SASP are capable of affecting HSCs within the bone marrow and drawing immune cells to areas of cellular senescence [71]. However, senescent cells then induce the opposite immunosuppressive state. This is first manifested by the correlation between senescence and the increased abundance of MDSCs, Tregs and anti-inflammatory M2 macrophages (Mreg/M2c) in both the bloodstream and tissues [71] and the significantly more pronounced suppressive effect of Tregs [72]. GM-CSF [73] and the chemokines CCL2 and CXCL2 [74, 75] significantly contribute to the chemotactic and proliferative activities of Tregs and MDSCs. In addition, T cells in elderly septic patients are more susceptible to apoptosis than those in younger septic patients are, which may lead to further impairment of proliferation, resulting in more severe lymphopenia. Analysis of the apoptosis scores of all the innate immune cell types revealed a uniform and significant increase in apoptosis scores with age and varying degrees of severe functional degradation (such as antigen presentation and phagocytosis) [72]. Notably, the formation pathway of extracellular traps in neutrophils significantly increases with age, and this phenomenon may be closely linked to the exacerbation of microcirculatory damage and unfavorable outcomes observed in elderly septic patients. In addition to inflammation, immune senescence is characterized by a significant reduction in the quantity and efficacy of immune cells, which further decreases with age; decreases in spleen weight and thymus weight; and notably, increases in the expression levels of markers associated with cellular senescence, including p16 and p21. As mentioned above, sepsis further weakens adaptive and innate immune functions that are weakened due to natural aging, inducing immunosenescence. These results indicate that the manifestations of sepsis are exacerbated in elderly individuals with sepsis, which also explains the poorer prognosis of sepsis in elderly individuals.

Restoring immune homeostasis in elderly sepsis patients is more difficult than in younger patients because of the immunosenescence that precedes the disease, which is reflected in higher mortality and poorer prognosis [76]. According to a longitudinal analysis involving 328 adult patients in the surgical intensive care unit (ICU) with sepsis, older patients (≥ 65 years) had a greater prevalence of comorbidities such as chronic kidney disease, coronary artery disease, and chronic lung disease than younger patients did (young, ≤45 years; middle-aged, 46-64 years). Additionally, elderly patients had a higher 30-day mortality rate, shorter ICU stays, greater likelihood of developing complications, lower likelihood of being discharged to nonhome destinations, and increased 12-month mortality rates. This phenomenon is closely linked to the notable deterioration of immune system functionality observed in the elderly population. Subsequent comparisons of elderly patients with younger patients revealed higher levels of immunosuppression, including lower lymphocyte counts [77], which are linked to an unfavorable prognosis in patients with sepsis [78]. Elderly patients with chronic critical illness (CCI) and sepsis present increased levels of proinflammatory biomarkers, notably sPDL-1 and IL-10. Nevertheless, it is imperative to underscore the dualistic nature of the function of IL-10 in sepsis: it is both proinflammatory and anti-inflammatory. This does not indicate that elderly sepsis patients inherently exhibit a heightened degree of immunosuppression. The extent of immunosuppression is contingent on the severity of the underlying disease [79]. This result aligns with the findings reported by Yende et al. [80], who reported that nearly 50% of elderly patients with sepsis have elevated PDL-1 levels within 12 months, indicating long-term immunosuppression.

Furthermore, as previously discussed, the intestines play a pivotal role in maintaining immune balance. Age-related changes in the intestinal microbiome are highly variable, with the effects of gradual deterioration of digestive function, including inflammation caused by aging, genomic instability, cellular (and mitochondrial) dysfunction, reduced protease homeostasis and epigenetic disorders, which further lead to chronic diseases, metabolic disorders and impaired gut-brain communication, as well as lifelong individual lifestyles, especially diet, leading to changes in the microbiota of the elderly body, the loss of dominant symbiotic flora, and replacement by group 2 symbiotic bacteria and pathogens, resulting in a decline in health in elderly individuals [81]. Age-related intestinal flora imbalances are a driving force in immune system homeostasis [82], and the increased virulence of intestinal pathogens leads to an increased infection rate of sepsis in elderly individuals [83]. As previously mentioned, elevated levels of GLP-1 have been observed to be associated with the severity of sepsis, inflammation, and high mortality in both intensive care unit (ICU) patients and those who have been discharged from the hospital. Some studies have shown that elderly patients (compared with younger patients) and elderly patients with chronic critical illness (compared with elderly patients with rapid recovery) exhibit persistently high levels of GLP-1 and low levels of albumin [28, 77]. These high levels of GLP-1 indicate a failure of metabolic homeostasis to return to normal. In addition, compared with those in young patients, the levels of anabolic markers are significantly lower in elderly sepsis survivors, while IGF-1 has the ability to maintain cell survival, as well as anti-inflammatory, antioxidant and antiapoptotic effects [84].

Although sepsis induces hyperinflammation and immunosuppression in the body through multiple mechanisms, elderly individuals exhibit immunosenescence and low immune system function, which may increase susceptibility to sepsis events and CCI or early death, and the likelihood of adverse outcomes is much greater than that of young people with sepsis [85].

## 3. Major Cellular and Molecular Mechanisms Involved in Immunosenescence

In both premature immunosenescence in septic patients and natural immunosenescence in elderly patients, alterations in immune cells are of paramount importance. Decreases in immune cell numbers and functionality due to different factors induce premature immunosenescence and accelerate the process of immunosenescence. Understanding exactly what changes occur in these cells and the mechanisms underlying these changes is essential to further clarify the causes of sepsis-induced immune senescence; here, we discuss the changes that occur in sepsis in three types of cells, representing intrinsic and adaptive immunity, as well as the characteristics of their senescence.

### 3.1 Neutrophils

Neutrophils are crucial elements of the body's innate immune system and act as frontline defenses against infections. The vulnerability of elderly individuals to bacterial infections is closely related to alterations in neutrophils associated with aging. During the aging process, neutrophils exhibit decreased migration and phagocytosis abilities and are more susceptible to apoptosis upon activation, reducing their recruitment to the site of infection and their phagocytic activity against pathogens [86]. Toll-like receptor dysfunction [87] results in the suppression of the antimicrobial effect of neutrophils. Neutrophil functions, including interleukin (IL)-1β production, reactive oxygen species (ROS) production, neutrophil extracellular trap (NET) formation, phagocytosis, migration and bacterial clearance, are significantly suppressed in aged mice [88].

**Figure 2.**
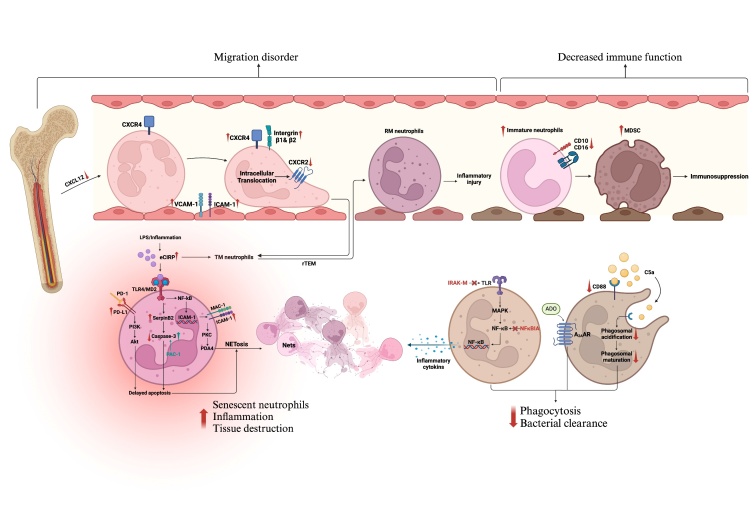
**Sepsis-induced premature neutrophil senescence**. In sepsis, increased TLR inhibitors (e.g., IRAK-M) and the NF-κB inhibitor NFκBIA impair bacterial clearance. Excessive C5a release inhibits phagocyte maturation via CD88, weakening neutrophil antimicrobial function. ADO accumulation activates neutrophil A2ARs, which exert immunosuppressive effects. Increased CXCR4 and decreased CD62L indicate neutrophil senescence. Senescent neutrophils migrate more to inflammation sites, releasing excess ROS and NETs, which cause inflammation and organ damage. Elevated CIRP exerts DAMP effects, promoting inflammation and organ damage, while increasing SerpinB2 and coinducing delayed neutrophil apoptosis with PD-L1, further increasing ROS and NET production. CXCL12 downregulation increases neutrophil release, whereas upregulated β1 and β2 integrins, ICAM-1, VCAM-1, CXCR4, and CXCR2 internalization hinder migration, causing some neutrophils to return to the circulation from inflammatory sites, as indicated by high ICAM-1 and low CXCR1 levels, exacerbating inflammatory injury. The proportion of immature neutrophils increases during sepsis, with decreased CD10 and CD16 expression, indicating that these cells exert immunosuppressive effects similar to those of MDSCs. (TLR, Toll-like receptor; NF-κB, nuclear factor-κB; C5a, complement component 5a; ADO, adenosine; A2AAR, A2A adenosine receptor; CXCR4, CXC receptor 4; ROS, reactive oxygen species; NETs, neutrophil extracellular traps; CIRP, cold-inducible RNA-binding protein; DAMPs, damage-associated molecular patterns; PD-L1, programmed cell death ligand 1; CXCL12, CXC ligand 12; ICAM-1, intercellular adhesion molecule-1; VCAM-1, vascular cell adhesion molecule-1; CXCR2, CXC receptor 2; CXCR1, CXC receptor 1; MDSC, myeloid-derived suppressor cell).

Studies on sepsis animal models and clinical studies performed on patients have shown dysfunction of neutrophils, including impaired bacterial scavenging and a reduced ability to recruit these cells to infected tissues [89]. Toll-like receptors (TLRs) are integral for identifying pathogens and triggering the subsequent secretion of antimicrobial peptides, proinflammatory cytokines, and chemokines. Additionally, they promote the production of ROS by stimulating the NF-κB and MAPK signaling cascades [90]. However, increased expression of TLR signaling inhibitors (e.g., IRAK-M) and the NF-κB inhibitor NFκBIA has been found in patients with sepsis [91]. Impaired neutrophil phagocytosis is also observed in patients with sepsis [91] due to excessive C5a release, which inhibits CD88 expression. By disrupting the maturation of phagocytes, C5a can also inhibit the antimicrobial function of neutrophils [51]. Evidence shows that extracellular adenosine accumulates in the metabolic stress zone of sepsis and exerts an immunosuppressive effect by engaging with and activating A2a adenosine receptors (A2aRs) coupled to G proteins on the neutrophil surface (Fig. 2) [92]. This results in a decrease in neutrophil degranulation, impaired chemotaxis, a diminished capacity for bacterial ingestion and killing, and delayed neutrophil apoptosis [93]. However, recent studies have shown that A2aR can prevent neutrophil aging and promote vitality in vitro and in a mouse model of sepsis. Activating A2aR in neutrophils from healthy individuals can delay cell aging, inhibit cell death, and cause neutrophils to shift from the proinflammatory N1 phenotype to the anti-inflammatory N2 phenotype. In contrast, in patients with sepsis, although neutrophil A2aR expression is significantly elevated, its level is not correlated with the aging process or N1/N2 polarization, suggesting that A2aR can regulate neutrophil aging only in healthy individuals but has no regulatory effect on the aging mechanism of neutrophils in sepsis [94]. However, studies have shown that in *Staphylococcus aureus* infections, *Staphylococcus aureus* can inhibit the antibacterial ability of human neutrophils by inhibiting the pentose phosphate pathway of A2aR [95]. As A2aR studies are based mainly on in vitro stimulation experiments, further modeling of the complex adenosine concentration dynamics in sepsis in vivo is needed.

Exposure to pathogens such as microorganisms in mice can lead to neutrophil senescence, as evidenced by increased CXCR4 expression and decreased L-selectin (CD62L) expression [96]. Cold-induced RNA binding protein (CIRP) is translocated from the nucleus to the extracellular space during sepsis. In addition to acting as a DAMP to promote inflammation and cause organ damage [97], eCIRP binds to TLR4 to upregulate SerpinB2 expression, which induces delayed neutrophil apoptosis and further promotes the accumulation of old neutrophils and the generation of ROS and NETs in sepsis [98]. The application of PAC-1, which is an apoptosis-inducing caspase-3 activator, can reverse this phenomenon [98]. Neutrophil PD-L1 expression is markedly upregulated in sepsis patients, and neutrophil apoptosis is inhibited by the PI3K/Akt pathway [99]. Delayed apoptosis is one of the main mechanisms by which prolonged neutrophil lifespan increases the ability of senescent neutrophils to produce NETs [100]. The ability of neutrophils to undergo senescence and antigen presentation induces the differentiation of Th1 cells, thereby exacerbating acute lung injury and shortening the survival period in patients with sepsis [101]. Therefore, senescent neutrophils are pivotal in the progression of sepsis.

As depicted in Figure 2, all stages of neutrophil migration are compromised during sepsis, including mobilization and liberation from the bone marrow, as well as subsequent processes such as rolling, adhesion, and migration. During sepsis, the downregulation of CXCL12 causes increased release of neutrophils into the peripheral circulation [102]. Neutrophils respond to pro-inflammatory mediators by upregulating β1 and β2 integrins, causing an increase in neutrophil granulocyte margination; these adhesion molecules engage with increased endothelial ICAM-1/VCAM-1 [103], enabling robust adhesion to endothelial cells and inhibiting subsequent extravascular neutrophil migration [103]. After sepsis, CXCR2 on the surface of neutrophils undergoes intracellular translocation, leading to the inhibition of neutrophil migration, and G protein-coupled receptor kinase (GRK) plays an important role in this process [104]. Consequently, neutrophil migration fails, and consistent with reduced chemokine receptor expression, septic patients exhibit significantly fewer neutrophils migrated to d3-4-stimulated pores [105]. Together with delayed neutrophil apoptosis, these effects lead to a massive accumulation of neutrophils in the vascular endothelium, resulting in a dysregulated immune response at the site of infection and a disruption of the body's ability to control the infection. Under normal circumstances, senescent neutrophils enter the bloodstream and migrate back to the bone marrow, where they are eliminated by macrophages [106]. While CXCR4 aids in the trafficking of aged neutrophils back to bone marrow for clearance, in inflammatory diseases such as sepsis, aged neutrophils in the circulation immediately recognize the inflammatory signal and quickly navigate to the site of inflammation, thus serving as the first line of defense in acute inflammatory responses. These neutrophils release excessive ROS and NETs [107], which contribute to inflammation and organ damage during sepsis. In addition, there is a phenomenon involving the reverse migration of neutrophils from the site of inflammation (i.e., the return of neutrophils from the site of inflammation to the circulation) [97], which is characterized by an ICAM-1^high^CXCR1^low^ phenotype [108]. Several mechanisms are involved, and a change in the concentration of the chemokine CXCL1 is critical for reversing neutrophil migration [108]. Substantial evidence indicates that, in diverse inflammatory environments, disruption of the CXCL1 gradient leads to the dysregulation of neutrophil migration and subsequent pathology [109, 110]. Inflammation leads to the destruction of endothelial junctions, and chemokines (such as CXCL1) leak from the circulation into the perivascular space, destroying the original chemotactic gradient and creating a "chemotactic repulsion" effect that directly promotes the reverse migration of neutrophils into the circulation [111]. CXCL1 binds to CXCR2 and, at high concentrations, can be transformed into a chemotactic exclusion signal. Like CXCL8, CXCL1 jointly regulates the direction of neutrophil migration [112]. Consequently, the aberrant distribution of the chemokine CXC in the context of sepsis has the potential to amplify neutrophil chemotaxis disorders, thereby becoming a pivotal chemokine molecule for reverse migration. Impaired autophagy in endothelial cells (ECs) is a marker of aging that induces excessive neutrophil infiltration in various acute inflammation models [113, 114]. Targeted transcriptome analysis and immune-fluorescence staining have revealed an increase in the level of the chemokine CXCL1 on ECs in vivo that was stimulated by the expression of the progerin protein [115], indicating that senescent ECs can induce excessive neutrophil trafficking and neutrophil-dependent vascular leakage. Moreover, these findings provide direct evidence that senescent ECs can drive dysregulated immune cell trafficking. In cases of acute chronic liver failure (ACLF), it has been shown that neutrophil infiltration can be significantly ameliorated by silencing CXCL1, which reduces ROS levels and hepatocyte apoptosis, thereby reducing inflammation and liver injury [116]. Whether this therapeutic approach can be applied to patients with sepsis requires further discussion.

Although definitive evidence for senescence markers (e.g., p16, p21, and p53) in prematurely aged neutrophils is lacking, β-galactosidase (β-Gal) staining has been observed in murine models of articular damage [117]. Senescent neutrophils exhibit elevated H2AX Ser-139 phosphorylation, a DNA damage and senescence biomarker [118], alongside increased NETosis induction and increased transcription/protein levels of PAD4 and Elane (azurophilic granule) [119]. Increased PAD4 is correlated with protein citrullination, gene suppression, chromatin depolymerization, and NET formation [120, 121]. The deficiency of grancalcin, a penta-EF-hand calcium-binding protein that is abundant in macrophages and neutrophils, reduces the neutrophil response to LPS-induced endotoxin shock [122]. Senescent neutrophils release elevated grancalcin in aged rodent bone marrow [123], and juvenile mice injected with recombinant grancalcin show binding of grancalcin to angiogenin-clusterin-B2 receptor, suppressing osteogenesis while promoting adipogenesis in marrow stromal cells and driving skeletal aging [123]. As sepsis progresses, an increasing number of Siglec-F+ neutrophils, which produce IL-10 and contribute to a suppressed immune response in the spleen, are observed in the spleens of mice. This increase is associated with a decrease in proinflammatory cytokine expression, a reduction in T lymphocyte functionality, and diminished activity among both CD4+ and CD8+ T lymphocyte subsets [124]. However, research on this phenomenon has focused only on the immunosuppressive effects of neutrophils in the spleen, and the systemic effects remain unclear.

Patients with severe burns exhibit persistent systemic inflammation accompanied by changes in the influx of immature neutrophils, T-cell subsets and cytokines [125]. Elevated levels of immature neutrophils have been linked to an increased risk of early death in sepsis patients [126]. Following the initiation of septic shock, there is a shift from an initial exaggerated neutrophil function characterized by a proinflammatory response to a state of immunosuppression. In septic shock patients, a greater incidence of immature neutrophils with a CD10dimCD16dim phenotype is observed, along with reduced expression of CD10 and CD16 on granulocytes [126], which are also characteristic of immature myeloid cells [127], whereas circulating immature neutrophils have a weaker role in supporting innate immune defenses in sepsis [128]. Given the similarity in phenotype and the observation that early MDSCs do not express immunosuppressive properties, further distinguishing MDSCs from immature neutrophils in sepsis is imperative. While the potential value of LOX-1 as a specific marker for MDSCs has been reported, further cohort verification is necessary because of the lack of clinical evidence [129]. Septic neutrophils also exert a suppressive effect on other immune cells, especially T cells. An elevated proportion of CD16^dim^ immature granulocytes in deteriorating patients is associated with a decrease in the proportions of CD3 and CD4 T-cell lymphocytes [126]. During episodes of intense inflammation, mature neutrophil subsets that enter the bloodstream can suppress the activation of T cells. This inhibition is facilitated by the specific delivery of hydrogen peroxide (H_2_O_2_) to the immune synapse in a process dependent on Mac-1 (comprising CD11b and CD18). H_2_O_2_ has a suppressive effect on T cells via multiple pathways, such as alterations in the expression of surface thiols and oxidation of the actin-depolymerizing protein cofilin [130, 131]. These results all demonstrate that neutrophils themselves undergo changes that lead to dysfunction during sepsis and can further induce immune system dysfunction.

These findings indicate that delayed neutrophil apoptosis and the generation of an inhibitory neutrophil subset during sepsis are key contributors to sepsis-induced early immunosenescence.

### 3.2 Macrophages

As specialized components of the innate immune system, macrophages are distinguished by their prolonged lifespan and phagocytic capabilities. Together with neutrophils, they act as first responders to infections. Macrophages play a pivotal role in identifying, phagocytizing, and breaking down cellular remnants and pathogens. They initiate adaptive immunity by presenting antigens to T cells and prompting other antigen-presenting cells to produce costimulatory factors. A crucial factor in the phenomenon of immunosenescence is the gradual loss of macrophage function. Senescent macrophages exhibit altered metabolism and immune function [132]; reduced levels of autophagy; modified cell polarization; diminished phagocytosis and antigen presentation; and impaired functions such as migration, infiltration and recruitment. These changes contribute to the development of diseases, including sepsis, across multiple systems throughout the body.

In septic mice, the activation of macrophages has been linked to cellular senescence, inflammatory signaling pathways such as the NF-κB pathway, and reactions to PAMPs. In these mice, a notable increase in the number of macrophage aggregates is correlated with cellular senescence, lymphocyte activation and lymphocyte functions, including adhesion and proliferation [101].

**Figure 3.**
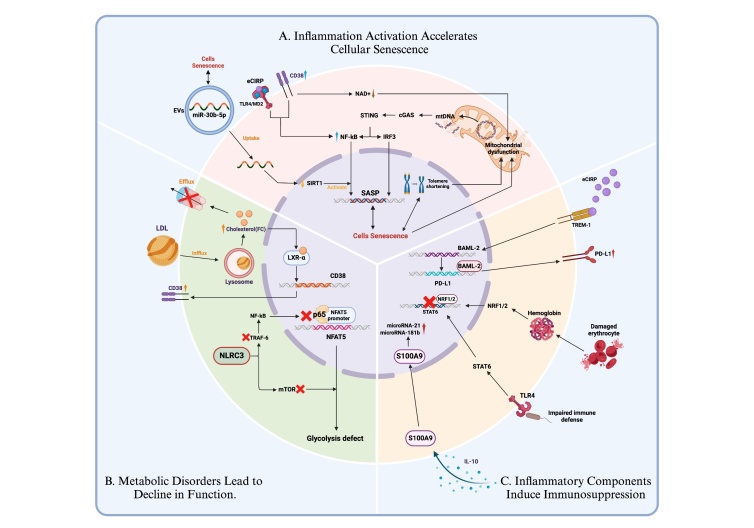
**Sepsis-induced premature macrophage senescence**. (**A**) Macrophage mitochondrial dysfunction in sepsis results in the release of mtDNA, which is recognized by cGAS/STING, activating the NF-κB and IRF3 signaling cascades to increase SASP synthesis and exacerbate inflammatory dysregulation. miR-30b-5p in SEN-EVs downregulates SIRT1, activating the NF-κB pathway to further promote SASP synthesis and accelerate cellular senescence. EVs in senescent and extracellular microenvironments transmit senescence-related signals, including SASP signals, in multiple ways. (**B**) After LPS activation, macrophages secrete more CD38, leading to impaired NAD+ synthesis and depletion. Cholesterol accumulation further depletes NAD+ through LXR-α activation, promoting macrophage senescence and dysfunction and accelerating immune senescence and immunosuppression. CD38 activation also enhances NF-κB signaling, promoting inflammatory gene expression. NLRC3 impairs immune and metabolic functions in immune-tolerant macrophages by inhibiting mTOR and TRAF6 activation and affects glycolysis and inflammatory factor synthesis by preventing NF-κB p65 and NFAT5 binding. (**C**) Increased macrophage processing of sRBCs during infection activates NRF1/NRF2 to inhibit STAT1, leading to immunosuppression. Elevated eCIRP in sepsis promotes PD-L1 expression via the TREM-1 pathway, inducing macrophage endotoxin tolerance and exacerbating immunosuppression, accelerating death. HLA-DRlow/S100Ahigh monocytes are associated with advanced sepsis and immunosuppression. S100A9 upregulates microRNA-21 and microRNA-181 in response to IL-10, promoting S100A9 trans-nucleation from membranes, increasing MDSC proliferation, and leading to immunosuppression. (mtDNA, mitochondrial DNA; NF-κB, nuclear factor-κB; IRF3, interferon regulatory factor 3; SASP, senescence-associated secretory phenotype; SEN-EV, senescent cell-derived extracellular vesicle; SIRT1, silent information regulator 1*;* EVs, extracellular vesicles; LPS, lipopolysaccharide; LXR-α, liver X receptor α; NLRC3, NLR Family CARD Domain Containing 3; mTOR, mammalian target of rapamycin; TRAF6, TNF receptor associated factor 6; NFAT5, nuclear factor of activated T cells 5; sRBC, senescent red blood cell; NRF, nuclear respiratory factor; STAT1, signal transducer and activator of transcription 1; eCIRP, extracellular cold-inducible RNA-binding protein; TREM-1, triggering receptor expressed on myeloid cells-1; BMAL2, basic Helix-Loop-Helix ARNT like 2; PD-L1, programmed cell death ligand 1; MDSC, myeloid-derived suppressor cell).

As discussed in the text above, telomere shortening occurs in the context of sepsis. Dysfunction of telomeres, resulting from either excessive erosion or compromised telomerase function, has been demonstrated to induce cellular senescence and contribute to the aging process at the organismal level [133]. In the context of respiratory tract infections, mice exhibiting dysfunctional telomeres exhibit increased susceptibility to respiratory virus infections. An aging-like phenotype has been identified in macrophages from mice with genetic deletion of telomerase RNA (Terc-/-), characterized by shortened telomeres, increased levels of the senescence-associated markers p16 and p21, and the secretion of aging-related inflammatory cytokines (Fig. 3).

These senescent macrophages release mitochondrial DNA due to mitochondrial dysfunction and are detected by the cell surveillance system (mainly the cGAS/STING system). This recognition initiates the NF-κB and IRF3 signaling cascades, enhancing the synthesis of inflammatory cytokines and IFNβ and thus exacerbating inflammatory dysregulation [134]. The administration of senolytics, which are compounds that eliminate senescent macrophages, has been shown to effectively enhance mitochondrial integrity and reduce the STING-mediated inflammatory pathway following treatment. This, in turn, has been shown to protect old Terc-/- mice from respiratory tract infections. However, it is important to note that these findings are limited to specific research subjects and the research context. Nevertheless, these findings underscore the notion that telomere shortening plays a pivotal role in the initiation of cellular senescence. This highlights the potential for developing treatments that aim to improve mitochondrial integrity or block the STING pathway to address inflammatory diseases [135]. In addition, senescent cell-derived extracellular vesicles (SEN-EVs) carry miR-30b-5p, which can activate the NF-κB pathway by directly downregulating SIRT1, and SIRT1 has anti-inflammatory properties [136]. These effects activate macrophages to induce chronic inflammation and further promote cell senescence, providing a novel explanation for the inflammatory process underlying the SASP [137]. The use of SIRT 1 agonists and miR-30b-5p antagonists both improved the inflammatory function-related processes of SEN-EVs, which clearly shows that this process can be a target for therapeutic modification and has therapeutic potential. In addition, EVs within senescent and extracellular microenvironments can convey aging-related signals, such as SASP signals, in an autocrine, paracrine and endocrine manner [138]. However, the specific role of SEN-EVs in immune senescence in sepsis needs to be further verified.

Following LPS activation, macrophages produce and secrete the NADase enzyme CD38 into the extracellular milieu. Nicotinamide adenine dinucleotide (NAD) is a coenzyme for redox reactions, making it central to energy metabolism. NAD is vital for maintaining redox balance and is indispensable for cellular functions such as signaling and DNA repair [139]. These cellular processes and functions are essential for maintaining tissue and metabolic homeostasis and healthy aging. However, the upregulation of CD38 in an inflammatory context leads to impaired NAD+ synthesis and subsequent depletion [140]. Cholesterol accumulation in bone marrow-derived macrophages (BMDMs) with defective cholesterol efflux under metabolic and genotoxic stress can deplete macrophage NAD+ through liver X receptor (LXR)-α activation and upregulate CD38 transcription [141]. A reduction in NAD+ levels triggers senescence and impairs macrophage function, resulting in the expression of markers indicative of senescence and promoting the progression of immunosenescence and immune-suppression [132, 141]. In addition, CD38 activation can further amplify NF-κB signaling. The transcription factor NF-κB is presumably a central regulator of the SASP, orchestrating the expression of the majority of proinflammatory genes within senescent cells [142], indicating a role in macrophage senescence. Notably, the development of cellular senescence and its secretory phenotype appear to be metabolically demanding, and low NAD+ during aging may inhibit the development of cellular senescence, including the SASP [143]. These observations highlight the complexity of the link between NAD+ metabolism and cellular aging, a link that still needs to be further characterized on the basis of the above observations. Therapeutic approaches to supplement NAD+ have been validated in some aging cells and diseases [144-146], but the use of these treatments in the context of sepsis remains uncertain.

Immunosuppression is also an important manifestation of macrophage senescence in sepsis, further exacerbating the progression of sepsis. Macrophage processing of senescent red blood cells (sRBCs) increases dramatically after *Klebsiella pneumoniae* infection in the lung. Stress erythrophagocytosis activates NRF1 and NRF2 to inhibit the STAT1 response. Defects in STAT1 signaling lead to an immunosuppressive phenotype, resulting in increased bacterial proliferation outside the lung and decreased survival of septic mice [147]. Furthermore, sepsis triggers increased secretion of eCIRP, which in turn stimulates the expression of the circadian gene BMAL2 via the TREM-1 pathway. BMAL 2 binds to its promoter to promote the transcription of programmed cell death receptor-1 (PD-L1), which induces endotoxin tolerance in macrophages, causing immunosuppression and accelerating sepsis death [148].

During sepsis, macrophages exhibit reduced expression of the MHC class II molecule HLA-DR, impairing antigen recognition and presentation. Low monocyte HLA-DR expression, which has been suggested as a major hallmark of immunosuppression [149, 150], is linked to altered monocyte function, decreased proinflammatory cytokine release, increased susceptibility to secondary infection and increased mortality rates among patients in critical conditions [66]. HLA-DRlowS100Ahigh monocytes are significantly enriched and are associated with late sepsis and immunosuppression [151]. S100A9+ monocytes, similar to monocytic MDSCs [152], strongly suppress CD4+ T-cell function by promoting MDSC expansion and function. Inhibition of S100A9 can reactivate CD8+ T-cell-driven antitumor immunity within lymphomas and mitigate the inhibitory effects of MDSCs in the context of sepsis [153]. Intracellular S100A9 increases signal transduction and transcription factor assembly in a manner that depends on IL-10, upregulating the expression of immunosuppressive mediators such as microRNA-21 and microRNA-181b, thereby promoting the proliferation of MDSCs and contributing to immunosuppression [154]. Signaling mediated by IL-10, coupled with reduced expression of the long noncoding RNA Hotairm1 (lncRNA Hotairm1), promotes the translocation of S100A9 from the cellular membrane to the nucleus [155], enhancing its immunomodulatory effects and the immunosuppressive function of phagocytes and MDSCs [151].

Metabolic reprogramming also plays a role in immunosuppression. Stress conditions prompt a shift in the energy production pathway from oxidative phosphorylation (OXPHOS) to glycolysis in monocytes/macrophages, which is crucial for the rapid release of proinflammatory cytokines. The acute phase of sepsis is characterized by immunosuppression, which manifests as a decrease in the glycolysis rate, oxygen consumption and fatty acid transport during the sepsis immune tolerance period [156]. The CARD domain within the NOD-like receptor family, specifically NLRC3, is linked to impairments in glycolytic processes in monocytes and macrophages from both septic patients and immunosuppressed mice [157]. In the context of sepsis, monocytes from 35 patients with bacterial sepsis presented significantly elevated NLRC3 levels compared with those from controls. The expression of HLA-DR and TNF-α was found to be inversely related to NLRC3 levels, with HLA-DR serving as an indicator of immunosuppression [66]. NLRC3 may disrupt both the immunological and metabolic functions of immune-tolerant macrophages by suppressing the activation of mTOR and TRAF6, which are coinducers of NF-kB translocation. Another transcription factor, nuclear factor of activated T cells 5 (NFAT5), which has structural similarities with the DNA-binding domain of NF-kB and contains two NF-kB consensus binding sites, is crucial for the regulation of glycolytic metabolism. By preventing the binding of NF-kB p65 and NFAT5, NLRC3 further modulates the transcription of genes associated with glycolysis and the synthesis of proinflammatory cytokines within immunosuppressive macrophages. These observations indicate that elevated NLRC3 levels in monocytes are correlated with the onset of sepsis immunosuppression, revealing that NLRC3 is an inducer of the hyperreactivity of monocytes and macrophages during sepsis.

**Figure 4.**
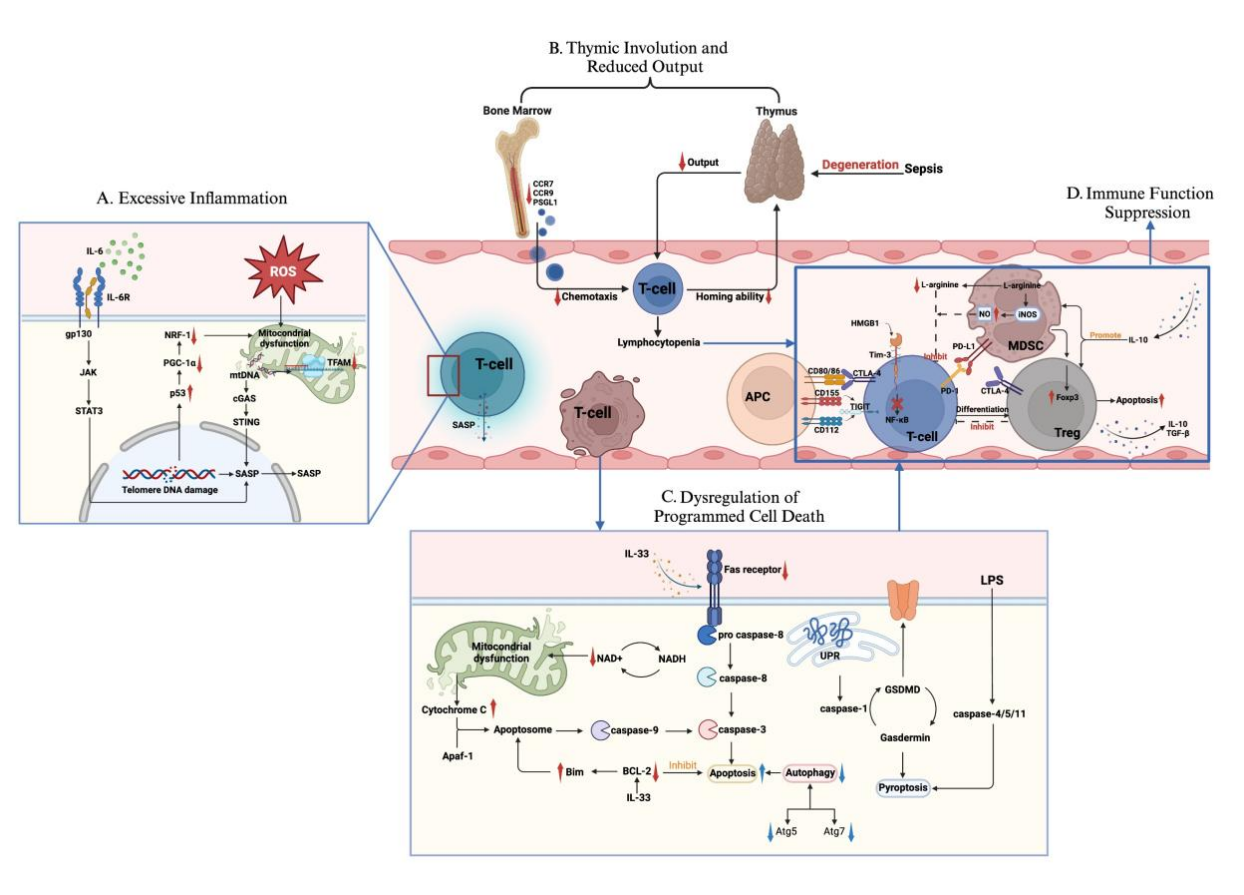
**Sepsis-induced premature T-cell senescence**. (**A**) Increased ROS levels in sepsis cause telomeric DNA damage and DDR, inhibit PGC-1α and NRF-1, and induce mitochondrial dysfunction in T cells. Released mtDNA activates cGAS and STING, increasing SASP production and inflammation and accelerating T-cell senescence. Reduced TFAM gene transcription leads to increased mtDNA accumulation, further accelerating senescence. A weakened NAD+/NADH balance exacerbates mitochondrial dysfunction. IL-6 binding to IL-6R triggers abnormal gp130/JAK/STAT3 signaling, causing multiorgan inflammation and senescence. (**B**) TPs have reduced migration to the thymus due to decreased CCR7 and CCR9 expression and lower P-selectin glycoprotein ligand 1, affecting T-cell maturation. Sepsis-induced thymic degeneration decreases thymic output and increases CD4+ T-cell senescence, exacerbating lymphopenia and immunosuppression. (**C**) The extrinsic apoptosis pathway activates caspase-8 via the Fas/Fas ligand pathway, initiating the caspase-3 apoptotic cascade. In the intrinsic pathway, cytochrome C is released and binds to Apaf-1 to form apoptotic vesicles, activating caspase-9 and promoting caspase-3 activation. Decreased BCL-2 expression in sepsis exacerbates T-cell apoptosis. When the UPR fails to maintain cellular homeostasis, it shifts ERS to a proapoptotic response, increasing caspase-1 recognition and processing of GSDMD and triggering pyroptosis. LPS binding to human caspase-4/5 or murine caspase-11 also activates GSDMD, leading to pyroptosis. Defects in the autophagy-related genes Atg7 or Atg5 downregulate autophagy, impairing its protective effect on T cells. (**D**) In sepsis, PD-L1/PD-1 interactions inhibit T-cell stimulation and initiate apoptotic pathways. CTLA-4 competes with CD80/CD86 on APCs to inhibit T-cell activation. TIGIT binding to CD155/CD112 activates immunosuppressive mechanisms. HMGB1 binding to TIM-3 inhibits NF-κB signaling in CD4+ T cells. Elevated MDSC levels promote T-cell depletion via PD-L1/PD-1 and express high arginase levels, increasing L-arginine catabolism and inhibiting T-cell function. MDSCs also promote FOXP3+ Treg cell development, contributing to immunosuppression. Peripheral IL-10 suppresses T-cell proliferation and proinflammatory cytokine release, increasing Treg and MDSC numbers. (ROS, reactive oxygen species; DDR, DNA damage response; PGC-1α, peroxisome proliferator-activated receptor-γ coactivator 1 α; NRF-1, nuclear respiratory factor 1; mtDNA, mitochondrial DNA; SASP, senescence-associated secretory phenotype; TFAM, mitochondrial transcription factor A; ETP, early T progenitors; CCR7, CC receptor 7; CCR9, CC receptor 9; Apaf-1, apoptotic protease activating factor-1; UPR, unfolded protein response; ERS, endoplasmic reticulum stress; GSDMD, gasdermin-D; LPS, lipopolysaccharide; PD-L1, programmed cell death ligand 1; PD-1, programmed cell death 1; CTLA-4, cytotoxic lymphocyte antigen 4; APC, antigen-presenting cells; TIGIT, T-cell immunoglobulin and ITIM; HMGB1, high mobility group box 1 protein; TIM-3, T-cell immunoglobulin and mucin domain 3; NF-κB, nuclear factor-κB; MDSC, myeloid-derived suppressor cell; NO, nitrogen monoxide; Treg, regulatory T cell).

### 3.3 T cells

T-cell senescence, which is characterized predominantly by a decline in the proliferative capacity and functionality of T cells and leads to immune defects and inflammation, may be a principal indicator of immunosenescence [158]. With age, there are systematic alterations in the composition and functionality of T cells. A marked reduction in the number of naive T cells leads to a significant change in both the quantity and characteristics of the T-cell population, consequently leading to a decline in the overall T-cell count with increasing age [159]. Compared with those in young people, the telomeres of T cells in elderly individuals are shorter, and T-cell receptor (TCR) lineage diversity is much more restricted [158]. When they become senescent, T cells lose their ability to secrete effector cytokines and are characterized by the expression of inhibitory molecules [160], which impairs the immune system's efficacy in confronting novel antigenic challenges. In addition, the function of senescent T cells decreases, and CD4+ and CD8+ T cells have a lower activation capacity. Ultimately, the immune system's ability to mount an effective response to novel antigenic stimuli is adversely affected. Secondary infections caused by T-cell damage strongly affect the prognosis and decrease the long-term survival rates of sepsis patients. After sepsis, the operational capacity of the adaptive immune system is suppressed overall. This can be attributed partly to the crucial function of intrinsic immune cells in early pathogen detection and response, as well as their role in orchestrating the adaptive immune system. Additionally, the decrease in various inhibitory elements and immune cell counts produced by septic organisms contributes to the weakening of the immune system and renders it incapable of functioning. Among immune cells, T cells are particularly impacted, and Figure 4 details these processes.

In sepsis and infections, multiple factors may directly induce T-cell senescence. T-cell senescence has been reported in infections with viruses such as HIV and HCV [161, 162] and bacteria such as Salmonella typhi [163], mainly due to telomere attrition and mitochondrial dysfunction caused by inflammation and stress [164, 165] and the activation of senescence-related signaling pathways [166]. Excessive ROS generated during viral infection or inflammation due to oxidative stress lead to telomere DNA damage and the DNA damage response (DDR) [165]. The DDR is a defining characteristic of cellular senescence and leads to cellular malfunction or apoptosis [37]. This process may suppress the expression of key regulatory proteins, including peroxisome proliferator-activated receptor-γ coactivator 1 α (PGC-1α) and nuclear respiratory factor 1 (NRF-1), resulting in mitochondrial dysfunction in T cells [167]. Importantly, in cases of cirrhosis and liver damage, the ability to filter out molecules derived from intestinal pathogens, such as lipopolysaccharide (LPS), is reduced because of damage to the liver's filtering ability, and pathogens persist. The resulting depletion or aging of T cells may be specific to HCV or microbial components [162]. Furthermore, premature T-cell senescence promotes systemic inflammation, which subsequently drives massive activation of bystander T cells, culminating in overall T-cell depletion and senescence [162]. However, most of the currently observed activation of bystander T cells occurs in cases of viral infection and tumors. Although these cells may be involved in inflammatory processes, further investigation is necessary to ascertain their potential significance in the context of sepsis [168, 169]. These results suggest that the effects of different diseases in the context of sepsis should be carefully considered. In addition, cytoplasmic mtDNA activates cGAS and STING protein activation, inducing the SASP, which further contributes to inflammation and T-cell senescence [170].

Upregulated mRNA expressions of oxidative damage markers and excessive mitochondrial DNA damage, as well as signs of poor mitochondrial quality, have also been found in patients with sepsis-associated AKI. This manifestation of mitochondrial damage is closely related to the LPS-induced downregulation of the mitochondrial transcription factor A gene TFAM [171]. TFAM is pivotal for the preservation of mitochondrial DNA and ATP production [172]. Defects in TFAM reduce the quantity of circulating CD4+ and CD8+ T cells, resulting in a significant increase in the mtDNA content within T cells and increasing T-cell senescence. Additionally, the equilibrium of oxidized to reduced forms of nicotinamide adenine dinucleotide (NAD+/NADH) in peripheral tissues is diminished, and even young mice show signs of mitochondrial dysfunction [172]. The administration of nicotinamide mononucleotide (NMN) to increase NAD+ levels can improve mitochondrial function, reduce mtDNA, and inhibit STING pathway activation, ultimately leading to the amelioration of motor capabilities, the prevention of inflammatory responses, and the deceleration of aging in CD8+ T cells [173].

IL-6 is a major proinflammatory factor. It is the only independent risk factor among all inflammatory indicators that predicts death from sepsis within 28 days. IL-6 is associated with mortality in sepsis patients [174]. In addition, IL-6 is also known to be a component of the secreted phenotype associated with aging and, together with cytokine signaling pathways such as the JAK/STAT pathway, promotes and maintains aging [175]. STAT signaling plays a major role in the expression of genes that control cell cycle progression, differentiation, proliferation, and apoptosis and contributes to aging and inflammation. Some studies have shown that inhibiting the JAK/STAT pathway can alleviate cellular senescence and age-related dysfunction [176]. IL-6 binds to its IL-6 R (IL-6 R α) to form the IL-6/IL-6 R complex, which causes the signal transduction receptor chain gp 130 to dimerize, thereby activating several intracellular pathways, including the JAK/STAT 3 pathway [177]. Abnormal gp130/JAK/STAT3 activation leads to disease-associated hyperinflammatory phenotypes in multiple organs, as well as signs of shortened lifespan and aging, and is also associated with increased cytotoxicity and Treg cell numbers in T-cell-restricted Lgp130 mice [178]. The JAK/STAT pathway may become a therapeutic target for the treatment of aging-related inflammatory diseases. In addition, TNFα initiates a positive autoregulatory loop through the persistently activated STAT pathway, inducing persistent DNA damage. TNFα also induces the secretion of cytokines, including IL-8, IL-6, IL-1α, and IL-1β, all of which are important components of the SASP [175]. Inflammation is associated with persistent DNA damage and increased ROS production during aging. It has been shown that cytokine signaling, persistent DDR, and ROS production are interconnected through feedback loops. Inhibition of the JAK/STAT pathway can alleviate cellular senescence and age-related dysfunction [175, 176]. In addition, the JAK/STAT signaling pathway is at the core of aging-related pathways and can interact with the IGF-1, P53-P21, and NF-KB signaling pathways to regulate aging and thus cause aging-related diseases [179-182]. Currently, a variety of drugs targeting the JAK/STAT pathway are available for the treatment of age-related diseases. In an etoposide-induced DNA damage senescence model, baricitinib was found to inhibit the JAK-STAT signaling pathway, reduce the number of senescent cells and the secretion of SASP factors and inhibit inflammation [183]. Since several families of JAKs and STATs exist, pathways composed of members of different families may be responsible for more specific tasks. When drugs are used for related treatments, it is necessary to consider which specific pathway the drug is specifically targeting to achieve a more specific therapeutic effect and avoid adverse outcomes.

In addition, overall degradation of the immune system is associated with a decrease in the number and functional degradation of immune cells. A reduction in T-cell numbers is a primary factor in immunosuppression caused by sepsis and significantly contributes to elevated long-term mortality rates among sepsis patients [58]. Studies have confirmed that patients experience significant lymphopenia after sepsis, and lymphopenia is associated with a worse outcome [24]. If cell counts do not return to normal, the risks of secondary infection, multiple organ failure, and mortality increase, and long-term immune dysfunction occurs [57, 184]. A reduction in immune cell counts coupled with a deterioration in immune function are typical manifestations of premature immunosenescence. This quantitative decrease consists of multiple components.

Thymic involution of an acute nature is a prevalent characteristic observed in infectious conditions and is a primary contributor to T-cell lymphocytopenia [185]. Sepsis-induced thymic degeneration leads to a decrease in thymic output and an increase in CD4+ T-cell senescence, promoting the progression of sepsis-related lympho-cytopenia and immunosuppression [19]. In addition, Kong et al. [63] reported that early T progenitors (ETPs) of hematopoietic stem cells exhibit decreased mRNA expression levels of the chemokine receptors CCR7 and CCR9, as well as P-selectin glycoprotein ligand 1. This reduction is associated with a compromised homing capacity both in vitro and in vivo, leading to impaired ability of bone marrow-derived progenitors to migrate to the thymus and consequently affecting T-cell maturation [63].

Another key factor contributing to the reduction in T-cell counts may be the promotion of programmed cell death. Apoptosis in T cells can be initiated through either the intrinsic mitochondrial pathway or the extrinsic death receptor pathway [186]. In the extrinsic apoptotic pathway, the engagement of the Fas/Fas-ligand pathway on T cells stimulates the activation of caspase-8, subsequently initiating caspase-3 and the cascade of apoptosis. Within the intrinsic pathway, cytochrome C is discharged from the mitochondria into the cytoplasmic space, where it complexes with apoptotic protease activating factor-1 (Apaf-1) to form apoptotic bodies. Caspase-9, which is abundantly expressed in patients with sepsis [186], is activated by apoptotic vesicles, subsequently promoting the activation of caspase-3 [11]. BCL-2 family proteins primarily inhibit apoptosis. In the CLP mouse model, a notable upregulation of the expression of cytochrome C, Bim, and the caspases -3, -8, and -9 has been reported, whereas BCL-2 expression was diminished, consequently facilitating T-cell apoptosis [187]. In addition, the unfolded protein response (UPR) normally counteracts lymphocyte stress. However, during sepsis progression, if the UPR cannot reestablish cellular equilibrium, the endoplasmic reticulum stress (ERS) response shifts to a proapoptotic mode; the key nuclear caspase-1, which recognizes and processes gasdermin-D (GSDMD), is upregulated, leading to pyroptosis. The noncanonical pathway involving the binding of cytoplasmic lipopolysaccharide (LPS) to human caspase-4/5 or murine caspase-11 triggers the activation of GSDMD and further leads to pyroptosis [11]. Notably, decreased autophagy induces apoptosis in T cells, and sepsis leads to the downregulation of autophagy in T lymphocytes. Atg7 or Atg5 are autophagy-related genes that play a positive role in maintaining peripheral lymphocyte populations [188]. In a CLP model of sepsis induction, electron microscopy revealed a reduction in autophagic vesicles within the spleens of T-cell-specific Atg7-deficient mice post-CLP, which presented increased mortality rates due to sepsis, increased T-cell apoptosis, and depletion of CD4+ and CD8+ T cells [189]. Thus, autophagy may protect T lymphocytes from apoptosis and immunosuppression triggered by sepsis. Moreover, IL-33 also attenuated T lymphocyte apoptosis and increased survival rates in a CLP model by decreasing Fas expression and increasing BCL-2 expression [190].

An immunosuppressive effect is observed during the initial phases of sepsis, which is mediated by the depletion of T cells through the interaction of the PD-L1/PD-1 axis [191]. The receptor programmed cell death 1 (PD-1, also known as CD279) is instrumental in controlling T-cell counts and their functional capabilities. When PD-L1 engages with its receptor PD-1, it delivers a coinhibitory signal that dampens T-cell stimulation and initiates the apoptotic pathway [192, 193]. Elevated levels of PD-1 expression on peripheral blood T cells correlate with reduced T-cell proliferation, a higher frequency of hospital-acquired infections, and an increased mortality rate among individuals with sepsis [13]. Other immune checkpoint molecules are also upregulated in sepsis. Cytotoxic lymphocyte antigen 4 (CTLA-4), an important immune checkpoint, joins the common ligand CD80/CD86 with the costimulatory factor CD28, which competitively binds to APC surface ligands to inhibit T-cell activation [194]. The T-cell immunoglobulin and ITIM (TIGIT) structural domains also activate immunosuppressive mechanisms through competitive binding to CD155/CD112. HMGB1 binds to TIM-3 in sepsis and suppresses the NF-κB signaling cascade within Tim-3-expressing CD4+ T cells, inhibiting T lymphocyte proliferation and the production of proinflammatory factors [195]. BTLA activation promotes T lymphocyte apoptosis in sepsis patients [196].

The increase in the number of immunosuppressive cells further weakens T-cell function. In sepsis patients, MDSCs are markedly elevated following disease onset and are responsible for the suppression of T-cell immunity through diverse mechanisms. The abundance of PD-L1-high MDSCs, especially polymorphonuclear (PMN)-MDSCs, is notably increased in sepsis, and these cells contribute to immunosuppression by promoting T-cell depletion via the PD-L1/PD-1 interaction during the initial phases of sepsis [191]. MDSCs expressing high levels of arginase and iNOS lead to increased catabolism of L-arginine, which decreases T-cell CD3ζ expression and prevents its upregulation of cell cycle regulators such as cyclin D3 and cyclin-dependent kinase 4 (CDK4). NO derived from L-arginine metabolism inhibits JAK3 and STAT564 in T cells, suppresses MHC class II expression, and induces apoptosis [197, 198]. The production of peroxides by direct contact between MDSCs and T cells leads to the nitration of TCRs and CD8 molecules. This modification disrupts specific peptide binding to T cells, causing them to become unresponsive to antigen-specific signals [198]. MDSCs also promote FOXP3+ regulatory T (Treg) cell development in vivo [199]. This evidence indicates that MDSCs further exacerbate immune-suppression in sepsis. During sepsis, CD4+ T cells can differentiate into regulatory T lymphocytes (Tregs) and suppress the immune response [200]. Tregs may mediate the onset of septic immunosuppression through a variety of pathways, as evidenced by the upregulation of Foxp3 by Tregs to regulate T-cell differentiation [201, 202] and by the negative costimulation of receptors such as CTLA-4 [11] and Toll-like receptor expression, enhancing their immunosuppressive function. Tregs are capable of triggering apoptosis in effector T cells (Teffs), autoreactive thymic T cells and monocytes via the Fas/FasL signaling mechanism and the TGF-β1 axis. Additionally, Tregs secrete suppressive cytokines such as IL-10 and TGF-β [203], which directly or indirectly target various elements of the immune system, leading to a reduction in the quantity and function of immune cells and the onset of immunosuppression. Notably, IL-10 secreted by monocytes/macrophages and Th2 cells also inhibits T-cell proliferation, suppresses proinflammatory cytokine release, and increases the abundance of Tregs and MDSCs [204, 205].

## 4. Therapeutic Strategies Targeting Immuno-senescence in Sepsis

Senescent immune cells accelerate solid organ senescence and thus promote systemic senescence [50]; thus, the key to slowing aging is to stop the process of immune aging, i.e., to increase the number and activity of immune cells. In normal aging populations, some success has been achieved in slowing the aging process by improving lifestyle through the use of anti-inflammatory agents, antioxidants and senescent cell scavengers. We have already discussed the mechanism by which premature immunosenescence occurs during sepsis. Related therapeutic measures will be described next in terms of those that target immunosenescence and those that target subsequent immunosuppression (Table 1).

**Table 1:** Therapeutic strategies and drug applications for immunosenescence in sepsis.

Mechanism	Compounds	Effects		In vitro	In vivo	Clinical trials				Ref
						I	II	III	IV	
**Restore HSC function**	—	Deplete my-HSCs to restore lymphangiogenesis, limit myeloid-driven inflammation and improve immune function in aged mice.		√	√					[206]
	**Rapamycin**	Restore HSC function, boost naive lymphocyte production, and augment the immune response to flu vaccines in mice.		√	√					[207]
**Restore thymus function**	rhGH with DHEA and metformin	Stimulate thymus growth and enhance immune function.		√	√	√				[208]
	**MSC**	Enhance thymic structure and function and promote regeneration in aged rhesus monkeys.		√	√					[209]
	**Tα1**	Increase T-cell counts and function, activate dendritic cells (DCs), boost natural killer (NK) cell activity, and enhance macrophage phagocytosis in septic patients.		√	√	√	√	√		[210, 211]
**Protect telomeres and mitochondrial DNA**	TA-65	Activate TERT expression to lengthen telomeres and restore function.		√	√					[212]
	**Small molecule inhibitors of PAPD5**	Stabilize TERC, lengthen telomeres, and restore function.		√	√					[213]
	**NMN, rapamycin, resveratrol, metformin and 78c**	Elevate NAD+, enhance mitochondrial function, lower mtDNA, and inhibit STING in septic mice.		√	√					[140, 173, 211]
**Inhibit the production of senescence marker SASP**	Metformin	Inhibit NF-κB and SASP to ameliorate age-related conditions.		√	√	√	√			[214]
	**Rapamycin**	Inhibit the mTOR pathway, help alleviate SASP and extend the healthy life and lifespan of sepsis mice.		√	√	√	√			[215]
	**cGAS and STING inhibitors**	Directly inhibit cGAS and STING to block the inflammatory pathways, preventing SASP in sepsis.		√	√					[216, 217]
	**The IL-1R antagonist anabolic acid and the anti-IL-6R antibody tolizumab**	Target specific SASP components to inhibit SASP.		√						[218]
	**Melatonin**	Block H2BK120 acetylation and SASP gene upregulation by interacting with PARP and CREB-binding protein.		√						[219]
	**PAC-1**	Activate caspase-3 to induce apoptosis and counteract NET overrelease from delayed neutrophil death.		√	√					[98]
**Reverse immunosuppression**	Treat with specific cytokines	IFN-γ	Enhance monocyte function in sepsis and counter immunosuppression by reducing IL-10, with a potential trade-off of increased PD-L1 impairing immunity.	√	√	√	√			[220-223]
		**GM-CSF**	Restore monocyte HLA-DR expression, enhance TNF-α release, and alleviate immunosuppression in sepsis patients.	√	√	√	√	√		[224, 225]
		**IL-7**	Suppress T-cell apoptosis, boost function, direct immune cells to infections, curb inflammation, preserve organ function, and elevate survival in sepsis mice models and patients.	√	√	√	√	√		[226, 227]
	Reduce MDSC counts	Gemcitabine and 5-fluorouracil	Selectively deplete MDSCs and Tregs, reduce immature bone marrow cells and enhance sepsis mice survival.	√	√					[198, 228]
		**GSK-J4202**	Inhibit KDM6A demethylase to inhibit Hotairm 1 transcription in sepsis mouse models and patients' MDSCs, relocate nuclear S100 A9 to the cytoplasm, prevent MDSC differentiation, and promote MDSC depletion.	√	√					[229]
		**ATRA**	Boost T-cell expansion and decrease MDSC levels in septic mice and reduce immunosuppression.	√	√					[230]
		**5-azacytidine and entinostat**	Inhibit the expression of MDSC CCR2 and CXCR2 to suppress the aggregation of MDSCs and improve the survival rates of sepsis mice.	√	√					[231, 232]
	Inhibit immune checkpoints	Yervoy	CTLA-4 inhibitors, initial immune checkpoint blockers, pose notable side effects and induce severe immunotherapy complications.	√	√	√	√	√	√	[233]
		**Anti-PD-1 antibodies**	Enhance T-cell function, survival, and bone marrow phagocytosis in sepsis patients, while ensuring high safety.	√	√	√	√	√		[234-238]
	Inhibit lymphocyte apoptosis	AC-YVAD-CMK	Specific inhibition of caspase-1 reduces NLRP1 inflammasome expression, and suppresses sepsis, inflammatory cytokine secretion and leukocyte aggregation in septic shock mouse models.	√	√					[239-242]
		**Ethyl pyruvate**	Block caspase-11 and Gsdmd activation to decrease apoptosis and mortality in septic mouse models.	√	√					[243]
		**Z-DEVD-FMK and AC-DMLD-CMK**	Inhibiting caspase-3 and GSDME improves lung tissue integrity and reduces mortality in sepsis.	√	√					[244]

### 4.1 Restoring Normal HSC Hematopoietic Function

HSCs play a pivotal role in the reconstitution of the hematopoietic system following insults such as infections or inflammatory conditions, and inflammation-induced proliferation leads to the accumulation of HSC DNA damage, which drives stem cell apoptosis or senescence [245], followed by irreversible depletion of functional HSCs and impaired recovery [7]. In addition, inflammation-associated stress hematopoiesis leads to impaired HSC functions; for example, HSC functions exhibit decreased posttransplant reimplantation potential and bone marrow-biased differentiation [206]. When my-HSCs were depleted via antibodies, bal-HSCs were found to restore lymphangiogenesis, limit myeloid-driven inflammation, and improve immune function in aged mice [206], indicating a role in reversing HSC senescence.

In addition, metabolism regulates HSC function during homeostasis and senescence. Homeostatic HSCs are dependent mainly on anaerobic glycolysis; hypoxia-inducible factor 1-α (HIF-1α) is also more stable in HSCs, and compared with normal HSCs, HIF-1α-deficient HSCs are more sensitive to the cell cycle and ROS generation [246, 247]. Increases in the mitochondrial membrane potential and OXPHOS and ROS levels during the phases of cell proliferation and senescence, as well as increases in cumulative ROS levels and the promotion of replicative stress and mitochondrial dysfunction, collectively compromise the long-term functionality of HSCs [245]. In contrast, recent studies have revealed that HSCs have the highest sphingosine kinase 2 (Sphk2) expression levels among hematopoietic cells and that Sphk2 is expressed predominantly in the nucleus. The deletion of the Sphk2 gene stimulates the HIF1-α-PDK3 pathway, which enhances the metabolic adaptability and functionality of HSCs, sustains their long-term self-renewal capacity, increases their regenerative ability and attenuates HSC senescence [248], providing a potential therapeutic target. Previous studies have revealed that rapamycin treatment restores HSC function, increases the generation of naive lymphocytes and enhances the immunological response to influenza vaccination in aged mice [207].

Because septic inflammatory stimuli initiate and accelerate HSC self-renewal and senescence, proper anti-inflammatory measures are both a priority and critical. This approach will be discussed in more detail below.

### 4.2 Restoring Normal Thymus Function

The thymus, a pivotal component of the immune system, is associated with the generation and maintenance of T cells. Severe thymic regression in patients with sepsis [249] leads to decreased T-cell output and function. Delaying or reversing the aging of the thymus may restore the body's immune function. In one study, nine aged healthy volunteers were given a combination of recombinant human growth hormone (rhGH), growth hormone (dehydroepiandrosterone (DHEA)) and metformin [208]. After 9 months, the volunteers had significantly less perithymic adipose tissue and a significantly greater lymphocyte/monocyte ratio. In addition, in terms of epigenetic age, the volunteers were approximately 1.5 years ‘younger’. These findings indicate that the thymus resumed growth, and that the body's immune system was strengthened. Treatment with mesenchymal stem cells (MSCs) significantly improved both the structural integrity and functional capabilities of the thymus in elderly rhesus monkeys, promoting thymus regeneration and enhancing thymus function [209]. In addition, pluripotent stem cells have been used to generate thymus tissue for possible transplantation to restore thymus function [250]. Administering thymosin α1 (Tα1) as part of sepsis treatment not only increases the number of CD4+ and CD8+ T cells and their functionality in individuals with lymphoma [210] but also stimulates DCs, augments natural killer (NK) cell activity, promotes macrophage phagocytosis, promotes HLA-DR in monocytes and inhibits the expression of PD-L1 [210, 211]. Lu et al. [251] have even suggested that partial reprogramming of immune cells may allow the reversal of cellular senescence, restoring them to a functional and youthful state.

### 4.3 Protecting the Integrity of Telomeres and Mitochondrial DNA

Sepsis-induced telomere attrition and telomere dysfunction are strongly associated with markers of aging, the onset of diseases associated with aging, and the progression of both inherited and acquired degenerative conditions, making the use of telomerase repair a promising therapeutic approach to counteract the effects of aging. Several small-molecule pharmaceuticals that activate telomerase reverse transcriptase (TERT) expression, including TA-65 and histone deacetylase inhibitors, have been identified [212]. Drugs that target TERC stability, such as PAPD5 small-molecule inhibitors, also increase telomere length [213], and pulsed telomerase activation therapies have exhibited some efficacy in the treatment of premature aging [212]. In addition, telomere damage can directly lead to damage and release of mitochondrial DNA and activation of inflammatory pathways. Supplementation of NAD+ with nicotinamide mononucleotide (NMN) improves mitochondrial function, reduces mtDNA, and prevents STING activation [173]. Several drugs, such as rapamycin, the autophagy inducer resveratrol, and metformin, also increase the NAD+/NADH ratio [252]. Since inflammatory CD38 upregulation directly leads to impaired NAD+ synthesis and depletion [140], direct inhibition of CD38 may be an effective approach. Most CD38 inhibitors are antibodies; for example, Darzalex (daratumumab) is used primarily to treat patients with multiple myeloma; however, CD38 antibodies do not inhibit intracellular CD38 [253]. In contrast, small molecule 78c therapy increases intracellular NAD+ levels and attenuates age-related metabolic dysfunction [254]. However, further validation and exploration of therapeutic options for CD38 inhibition are needed. In addition, anti-inflammatory measures are also important because inflammation directly increases CD38 levels. One effective strategy may be to increase mitochondrial heterogeneity by increasing the amount of mitochondrial wild-type mtDNA through the use of mitochondrial targeting strategies or genome editing to repair mutated mtDNA and eliminate mutated mtDNA [255]. Although it has been shown to be effective in in vitro cellular experiments and mouse models, the feasibility of this treatment needs to be further explored due to the complexity of the human system.

### 4.4 Inhibiting the Production of the Senescence Marker SASP

Inflammatory stimuli in sepsis not only cause direct tissue damage but also promote the senescence of a variety of immune-related cells. Associated substances promote SASP expression through inflammatory transduction signals such as the NF-κB and cGAS/STING pathways, and subsequently, the SASP activates senescence pathways in surrounding cells, creating a vicious cycle. In terms of anti-inflammatory treatments for immune-senescence, the main mechanism involves the suppression of the SASP. Indirect effects on SASP may be achieved by improving mitochondrial function. Metformin, aspirin, rapamycin, and ibuprofen are basic anti-inflammatory agents. The antidiabetic drug metformin ameliorates numerous conditions associated with aging across experimental models and in human studies. It inhibits several proinflammatory SASP factors by impeding the nuclear translocation of NF-κB [214]. Rapamycin and its derivatives, collectively referred to as 'rapalogs', mitigate the SASP through the inhibition of the mTOR pathway, which seems to extend both the health and lifespan of mice [215]. Because of the strong correlation between cGAS-STING and inflammation and aging, direct blockade of the inflammatory pathway with inhibitors of cGAS and STING may also prevent SASP production [216, 217]. In addition, the SASP can be inhibited by targeting specific SASP components; some agents with this mechanism include the IL-1R antagonist anabolic acid or the anti-IL-6R antibody tolizumab [218]. During inflammation, innate immune cells rapidly produce the highly proinflammatory classical class of eicosanoids to clear undesirable stimuli. However, after the stimulus is controlled, the same immune cells begin to produce lipids with reparative effects, such as SPMs, which mediate inflammatory abatement [2], illuminating the potential therapeutic role of SPMs. Melatonin can prevent H2BK120 acetylation and inhibit the upregulation of the SASP gene via poly (ADP) ribose polymerase and CREB-binding proteins [219]. In addition, delayed neutrophil apoptosis leads to excessive NET release, which can be reversed by PAC-1 activation of caspase-3 to promote apoptosis as well as by treatment with PD-1 antagonists [98].

### 4.5 Reversing Immunosuppression Induced by Immunosenescence in Sepsis

#### 4.5.1 Treatment with Specific Cytokines to Reverse Immunosuppression

IFN-γ promotes macrophage phagocytosis and pathogen elimination by activating antigen-presenting cells and monocytes through MHC I/II molecules. It enhances immune function by increasing lysosomal activity and promoting the release of inflammatory mediators such as TNF-α, IL-1, and IL-6. In some clinical studies, IFN-γ has the potential to improve monocyte dysfunction in sepsis patients by increasing mHLA-DR expression and TNF-α secretion [220] while reducing IL-10 expression, thus reversing immunosuppression [221]. However, IFN-γ induces regulatory T-cell apoptosis in tumors [222] and may upregulate PD-L1 to impair immunity [223], highlighting its dual role. Since IFN-γ is an inflammatory cytokine, the safety of its application requires further investigation.

GM-CSF is a hematopoietic factor that increases neutrophil and monocyte production, activates monocytes, and enhances immune function by improving phagocytosis and bactericidal capabilities. Research indicates that it can reestablish HLA-DR expression in monocytes from sepsis patients, promote TNF-α release, and reverse immunosuppression [224]. In randomized, double-blind clinical trials, GM-CSF treatment significantly increased mHLA-DR expression, restored immune function, and reduced mechanical ventilation, hospital stay, and intensive care unit (ICU) treatment durations [256]. Pinder et al. [225] reported that neutrophil phagocytosis in 10 patients in the GM-CSF group increased by more than 50%, whereas only 7 (44%) of 16 patients in the control cohort exhibited comparable effects, indicating the potential of GM-CSF to enhance immune cell function and decrease secondary infections in the context of sepsis.

IL-7, a cytokine with proinflammatory and antiapoptotic properties, is secreted by multiple cell types and serves as a growth factor for both T and B lymphocytes. Recombinant human interleukin-7 (rhIL-7) has been applied in the treatment of both idiopathic and acquired lymphopenia. Intervention with IL-7 notably increased survival rates in septic model mice and inhibited CD4+ and CD8+ T-cell apoptosis. It also increases T-cell functionality and facilitates the migration of immune effector cells to infection sites [226]. A phase II clinical study involving 27 patients who experienced septic shock and severe lymphopenia demonstrated that IL-7 therapy led to considerable increases in the counts of CD4+ and CD8+ T lymphocytes without producing an excessive inflammatory response or increased organ dysfunction [227]. These results confirm the beneficial effects of IL-7 treatment on lymphocyte counts and immune recovery in sepsis patients.

#### 4.5.2 Reducing the Number of Immunosuppressive MDSCs

MDSCs are pivotal in mediating immunosuppression during sepsis and significantly contribute to the deterioration of immune function in this context. Many studies have revealed the therapeutic potential of targeting MDSCs in cancer [230], with three main strategies: direct elimination, differentiation modulation, and migration inhibition. Chemotherapies such as gemcitabine and 5-fluorouracil can selectively deplete MDSCs and Tregs [257] and decrease the number of immature myeloid cells, which increases survival rates in mice [228]; notably, the counts of other immune cells are unaffected by these treatments [198]. In the context of sepsis, inhibiting the long noncoding RNA Hotairm1 with GSK-J4 [229] and targeting S100A9 with a peptone antibody can prevent MDSC differentiation and promote MDSC depletion [230]. Promoting the differentiation and maturation of MDSCs may also be a viable option. In a murine model characterized by LPS-induced immunosuppression, treatment with ATRA facilitates the recovery of T-cell proliferation and reduces the quantity of MDSCs, attenuating the immunosuppressive effects of sepsis [258]. Blocking the binding of chemokines to the corresponding receptors on MDSCs constitutes a strategic approach to impede MDSC migration [230]. Blocking chemokine receptors on MDSCs, such as CCR5, can inhibit their recruitment and improve survival in mouse models [231]. In addition, the use of low concentrations of a DNA methyltransferase inhibitor (5-azacytidine) and a histone deacetylase inhibitor (entinostat) inhibits the aggregation of MDSCs through the suppression of CCR2 and CXCR2 expression [232]. However, the applicability of these findings in the context of nontumour sepsis needs to be further investigated.

#### 4.5.3 Reducing the Number of Immunosuppressive Cells

Targeted Treg therapy is highly important in tumor therapy and has significantly improved outcomes, but the direct elimination of Tregs needs to be further studied in the context of sepsis. Tregs play a significant immunosuppressive role in sepsis, and their depletion is hypothesized to increase immune function and improve outcomes in sepsis patients. Neutralizing IL-10 or TGF-β, which are key in Treg differentiation, can alleviate immunosuppression in sepsis, reduce the ratio of Tregs to CD4+ T cells, and restore the number of CD4+ T cells in the spleen, potentially increasing survival [203]. However, CLP experiments in mice revealed that depleting Tregs with antibodies did not improve survival, and human septic shock data revealed higher Treg ratios and numbers in survivors by day 5 postonset than in those who died [259]. These findings indicate that Tregs play a protective role in sepsis progression. In a sepsis model with 95% Treg depletion, removal did not inhibit inflammatory responses or reduce early mortality, but Foxp3+ Tregs had a positive prognostic effect after 36 hours. Survival rates differ between Treg-depleted (5%) and Treg-active (25%) CLP models [260], indicating that indiscriminate Treg depletion may not improve patient prognosis. In the highly proinflammatory environment of an organism, the immunosuppressive effects of Tregs may also be inhibited, but these effects may be reversed as inflammation subsides. If the body does not stop the anti-inflammatory process, immune failure may ultimately occur. The timing of Treg reduction is crucial for preventing immune failure. Another study revealed that dying Tregs release adenosine triphosphate (ATP) and then rapidly convert ATP into adenosine, which suppresses T-cell function by binding PD-L1 [261]. Notably, blocking Treg SREBP pathways improved anti-PD-1 treatment responses without affecting Treg proliferation or function, limiting immune-related toxicity targeting Tregs [262]. Further experiments are needed to determine the effects of Treg-targeted therapies.

#### 4.5.4 Administration of Immune Checkpoint Inhibitors to Enhance Immune function

Targeted immune checkpoint therapies enhance resistance to infection and improve outcomes. CTLA-4 was the first immune checkpoint identified, and Yervoy was the first inhibitor approved; however, it has severe side effects [233]. CTLA-4 itself has normal physiological functions; notably, Yervoy recruits cell surface CTLA-4 to lysosomes for degradation, resulting in CTLA-4 deficiency in the human body and leading to serious immunotherapy-related adverse events. Therefore, attention has gradually shifted to PD-1 inhibitors, which have better safety profiles. Treatment with anti-PD-1 antibodies beginning 24 hours post-CLP-induced sepsis has been shown to alleviate the T-cell dysfunction associated with sepsis and increase survival rates [234]. In a murine CLP-induced sepsis model, similar improvements in T-cell functionality, myeloid major histocompatibility complex II expression, and survival were noted [235]. In vitro incubation with anti-PD-1 antibodies has also been shown to suppress apoptosis and promote interferon-γ secretion in blood CD8+ T cells derived from sepsis patients [236]; it also promotes the phagocytosis of myeloid cells among peripheral blood leukocytes isolated from these patients [237]. A phase I clinical trial demonstrated the safety of an anti-PD-L1 antibody [238]. High-dose anti-PD-L1 treatment notably increased the expression of mHLA-DR, and this increase was maintained for more than 28 d without increasing the levels of cytokines, including IL-6, IL-8 and IL-10. These results indicate that anti-PD-1 therapy is an effective intervention, but its safety and efficacy need to be further confirmed in phase II and III clinical trials. Additionally, TIM-3 knockdown in CD4+ T cells or systemically in sepsis models reduces immunosuppression-related mortality [195].

#### 4.5.5 Inhibiting Lymphocyte Apoptosis

Caspases are a group of proteases found in the cytoplasm of cells and are important mediators of programmed death (including apoptosis, pyroptosis and necrotic apoptosis). Targeting caspases can reduce cell loss and improve outcomes in the context of sepsis. In septic mice, the apoptosis rate of thymic and splenic lymphocytes, as well as the mortality rate, was significantly reduced by the administration of caspase inhibitors. In a murine model of septic shock, AC-YVAD-CMK, a specific caspase-1 inhibitor, demonstrated a protective influence on vital organs such as the liver, brain, and lungs [239-241] by reducing NLRP1 inflammasome expression and inhibiting inflammasome pathway-mediated sepsis in renal tubular epithelial cells, attenuating NLRP1 inflammasome-triggered secretion of the proinflammatory cytokines IL-1β and IL-18, sepsis-induced acute kidney injury, and histological damage to renal tissue, and inhibiting neutrophil and macrophage aggregation in renal tissue [242]. Blockade of the pathway involving gasdermin D (GSDMD), a downstream molecule of caspase-mediated scotomization, can prevent cell death. In LPS-induced endotoxemic mice, the administration of ethyl pyruvate suppressed the activation of caspase-11 and the subsequent cleavage of Gsdmd, reducing apoptosis and lethality [243]. The expression of caspase-3 and the N-terminus of GSDME was increased in the lung tissue of mice subjected to the CLP procedure, with structural disruption of the lung tissue and elevated levels of LDH, IL-6, IL-18 and IL-1β. The use of a specific caspase-3 inhibitor, Z-DEVD-FMK, and an inhibitor derived from GSDME, AC-DMLD-CMK, improved lung tissue conditions in the treated group of mice [244]. While agents targeting caspases have therapeutic potential, the effects on other cellular processes and challenges related to dosing and timing limit their clinical use, necessitating further research.

## Conclusion and Perspectives

In this review, we assessed the differences in immune responses during sepsis between populations with premature immunosenescence and natural aging, analyzed the main manifestations and mechanisms of several immune cells that represent both intrinsic and adaptive immunity, and finally proposed possible therapeutic interventions against premature immunosenescence aimed at improving immune function in sepsis patients and reducing the rates of secondary infections and adverse outcomes.

Previous studies have clearly shown the mechanisms and manifestations of immunosenescence and its impact on age-related diseases and human aging, but few studies have linked the immunosuppression observed in sepsis to immunosenescence. One reason for this may be that immunosenescence is a phenotype considered specific to elderly individuals, whereas sepsis affects a wide range of people of all ages. However, the proposed definition of premature senescence clearly indicates that immunosuppression in sepsis is the premature regression of immune organs, i.e., premature immunosenescence, that occurs in response to sepsis injury.

Although both premature immunosenescence and natural immunosenescence manifest as immune-suppression, the focus should be on the differences that exist. Unlike natural immunosenescence, which can only be slowed, premature immunosenescence in sepsis can be reversed or partially reversed with appropriate treatment. It has been observed that neutrophil apoptosis is delayed in sepsis patients, but neutrophil apoptosis is increased in elderly individuals [263], which facilitates antagonism of the inflammatory response and maintenance of homeostasis in vivo by eliminating senescent cells; notably, neutrophil performance in sepsis patients clearly does not correspond to this classical manifestation of senescence. Indeed, septic neutrophil apoptosis delays the progression of sepsis to immunosuppression, whereas neutrophil senescence in elderly individuals is a self-protective mechanism that delays immune decline.

There is no definitive evidence that immune cells express senescence markers such as senescence-associated beta-galactosidase (SA-β-gal) and the cyclin-dependent kinase inhibitors p21 and p16 in the context of sepsis. Identifying these factors could clarify whether immune cells become irreversibly senescent in sepsis or if premature immunosenescence due to sepsis can induce the production of such markers, providing new therapeutic options against sepsis. For example, senolytic drugs such as dasatinib and quercetin, which are being studied for their potential to slow aging by reducing p16 and SA-β-gal expression and improving physical function in aged mice [38], may be valuable for sepsis treatment. More research into the correlations and differences between sepsis and immunosenescence may provide better treatment options for sepsis patients and elderly individuals and advance our understanding of immunity and aging.
